# Voriconazole Composited Polyvinyl Alcohol/Hydroxypropyl-β-Cyclodextrin Nanofibers for Ophthalmic Delivery

**DOI:** 10.1371/journal.pone.0167961

**Published:** 2016-12-14

**Authors:** Xiaoyi Sun, Zhenwei Yu, Zhengyuan Cai, Lingyan Yu, Yuanyuan Lv

**Affiliations:** 1 Department of Pharmacy, Zhejiang University City College, Hangzhou, Zhejiang, China; 2 Sir Run Run Shaw Hospital, College of Medicine, Zhejiang University, Hangzhou, Zhejiang, China; 3 Second Affiliated Hospital, School of Medicine, Zhejiang University, Hangzhou, Zhejiang, China; Brandeis University, UNITED STATES

## Abstract

Voriconazole (VRC) incorporated in composited polyvinyl alcohol (PVA)/hydroxypropyl-β-cyclodextrin (HPβCD) blended nanofibers were produced via electrospinning for efficient ophthalmic delivery. The VRC loading capacity increased with increasing HPβCD content. The optimal solution for electrospinning consisted of 8% (w/v) PVA, 4% (w/v) HPβCD and 0.5% (w/v) VRC. The nanofibers exhibited bead-free average fiber diameters of 307±31 nm and VRC was released *in vitro* in a sustained manner. The VRC nanofibers were characterized by infrared spectroscopy (FTIR), thermogravimetric analysis (TGA), differential scanning calorimetry (DSC) and scanning electron microscopy (SEM). The proton nuclear magnetic resonance (^1^H-NMR) was used to analyze the molar ratio of HPβCD/VRC in the nanofibers. Compared with a VRC solution, the nanofibers significantly prolonged the half life, and increased the bioavailability of VRC in rabbit tears. No obvious signs of irritation were observed after application in the conjunctival sac. VRC nanofibers are promising for ophthalmic drug delivery and further pharmacodynamics studies are needed.

## Introduction

Fungal keratitis, a fungal infection of the cornea, is one of the most serious ocular fungal infections. With the extensive application of broad-spectrum antibiotics and corticosteroids, the incidence of fungal keratitis has increased recently. It has become the major cause of blindness caused by ocular fungal infections [1, 2]. The first-line therapy for patients with fungal keratitis includes topical antifungal agents alone or in combination with systemic antifungal agents. However, the lack of specificity of antifungal agents is the main obstacle to successful therapy [3].

Voriconazole (VRC), a triazole antifungal agent, is a lipophilic drug (LogP 1.65) with low aqueous solubility (0.5 mg/ml in distilled water). It possesses promising characteristics such as broad-spectrum activity, activity against resistant fungal species, good oral bioavailability, and acceptable tolerability. It is highly effective against various fungal isolates associated with keratitis [4, 5]. It is commercially available as oral and i.v. preparations. However, adverse effects including visual disturbances, hepatic abnormalities and interactions with concomitant medications (attributed to its role as both an inhibitor and substrate of CYP2C19, CYP2C9, and CYP3A4) make topical application an attractive route of delivery [6, 7]. Topical VRC can penetrate the cornea, and therapeutic concentration can be achieved in the cornea, aqueous humor and vitreous body [8, 9]. To date, no topical formulation for ocular use has become commercially available, although several attempts involving cyclodextrin based eye drop solutions and gels [10, 11], microemulsions [12], liposomes [13], and niosomes [14] have revealed the potential of VRC for topical ophthalmic administration.

Electrospinning is a versatile and cost-effective method for producing nanoscale fibers from polymer solution or polymer melts with the help of a very strong electric field. Nanofibers have received great attention in tissue engineering, enzyme immobilization and drug delivery systems in recent years [15, 16]. The release [17, 18] and solubility [19] of poorly water-soluble drugs can be enhanced in nanofibers because of the large surface-to-volume ratios, highly nanoporous structures, short distances for diffusion and amorphous state of loaded drugs. If nanofibers are delivered to the cul-de-sac of the eye, they are easily wetted and swell. Effective drug concentrations in the eye can be achieved because of prolonged drug retention and controlled drug release from nanofibers [20]. To date, VRC-loaded nanofibers for oral use have been prepared. However, the polymer poly (ε-caprolactone) insolubility and long degradation time of such nanofibers makes them inappropriate for ocular applications [21]. There is a need to develop a hydrophilic system for VRC delivery. To the best of our knowledge, electrospun nanofibers for ophthalmic applications have not been previously investigated. In the present study, HPβCD was used in the formulation to solubilize VRC [11]. The application of cyclodextrins in ocular inserts has been reported previously [22]. Passive ocular permeation could be facilitated by increased drug concentrations at the ocular surface. Polyvinyl alcohol, a biocompatible polymer, has been widely used in ocular delivery systems [23]. The rational combination of PVA and cyclodextrin has previously been shown to be an efficient delivery technique [24]. Here, VRC and HPβCD have been incorporated in PVA nanofibers. The characteristics of the fibers and *in vitro* drug release, biocompatibility and pharmacokinetics in rabbit tears were investigated. The VRC nanofibers are safe for ocular use and they can significantly increase the half life and bioavailability of VRC in tears.

## Materials and Methods

### Materials

Polyvinyl alcohol (PVA AH-26) was supplied by Zhejiang Changqing Chemical Co., Ltd. VRC was purchased from Huahai Pharmaceutical Co, Ltd. (Taizhou, China). HPβCD from Fengyuan Biotechnology Co., Ltd (Jiangsu, China) and cetonitrile (Merck, Darmstadt, Germany) of HPLC grade were used. Male New Zealand white rabbits weighting 2.5–3.0 kg were purchased from Zhejiang Academy of Medical Science (Hangzhou, China). The rabbits were bred in normal way and all the animal experiments were performed in full compliance with guidelines approved by the Animal Care Committee at Zhejiang University City College. All efforts were made to minimize suffering.

### Phase solubility studies

The solubility study was performed according to the method reported by Higuchi and Connors [25]. Excess VRC was mixed in an aqueous solution containing increasing amounts of HPβCD, which was agitated on a shaker at 37°C for 24 h. Then, the samples were centrifuged and the VRC contents were quantified using high-performance liquid chromatography (HPLC). The experiments were carried out in triplicate.

### Preparation of VRC nanofibers

To determine the optimal concentration of PVA for the electrospinning process, 6%, 8% and 10% (w/v) PVA were used to prepare blank nanofibers. Subsequently, varied amounts of HPβCD were added to the polymer solution to achieve the final concentration of 4%, 6% and 8% (w/v). The effects of cyclodextrins on nanofibers morphology were determined. Finally, drug loaded spinning solutions were prepared by mixing PVA solutions with VRC dissolved in a HPβCD solution at 50°C. The finally solutions consisted of 8% (w/v) PVA, 4% (w/v) HPβCD and 0.5% (w/v) VRC (F1), 8% (w/v) PVA, 6% (w/v) HPβCD and 0.7% (w/v) VRC (F2), or 8% (w/v) PVA, 8% (w/v) HPβCD and 0.8% (w/v) VRC (F3).

The mixed solutions were then placed into a 20 mL plastic syringe equipped with a flat-tipped stainless steel needle (d = 1.2 mm). An emitting electrode of positive polarity from a high-voltage DC power supply (DW-P303-1ACFD, Dongwen High Voltage Supply, Tianjin, China) was connected to the spinning solution. A grounded copper plate was used as a collector. The electrostatic field strength was fixed at 18 kV/20 cm. The feed rate of the solution was controlled to 0.5mL/h using a syringe pump (WZ-50C6, Smiths Medical Instrument Co., Ltd, Zhejiang, China). For the morphological study, the collection time was approximately 5 min, while, for the rest of the experiments, the collection time was 6 h. The collected nanofibers were placed in a vacuum oven overnight at 40°C to eliminate residual solvent.

### Characterization of VRC nanofibers

#### Thickness

The thickness was measured with a hand-held micrometer (Mitutoyo, Japan). The values were determined at five different regions of the film.

#### Scanning electron microscope (SEM)

The morphological appearance of the nanofibers was assessed using a scanning electron microscope (SEM, TM-1000, Hitachi, Japan). The average diameter of the fibers was evaluated using Image J software by measuring 100 fibers from the SEM images.

#### The proton nuclear magnetic resonance (^1^H-NMR) analysis

The proton nuclear magnetic resonance (^1^H-NMR) spectra were recorded at 400 MHz (Bruker, AVANCE III 400, Switzerland). 5 mg VRC, 10 mg PVA/HPβCD nanofibers or 10 mg VRC nanofibers were dissolved in 0.5 mL d6-DMSO.

#### Thermogravimetric analysis (TGA)

Thermogravimetric (TGA, TA Q50, USA) analyses were performed for VRC, HPβCD, PVA, PVA/HPβCD nanofibers and VRC nanofibers. TGA was conducted under nitrogen atmosphere by heating the samples from 50 to 600°C at the heating rate of 20°C/min.

#### Differential scanning calorimetry (DSC)

A differential scanning calorimeter (DSC, Q20, TA, USA) was used to investigate the thermal behavior of the VRC nanofibers. The DSC thermograms (equilibrated with an indium standard; each sample weighed 3–5 mg) were obtained during heating from 35 to 260°C at a rate of 10°C/min.

#### Fourier transform infrared spectrophotometry (FT-IR)

The chemical structures of VRC nanofibers and PVA/HPβCD nanofibers were characterized using a Fourier transform infrared spectrophotometry (FT-IR, Nicolet 6700, USA) with the accessories of attenuated total reflectance (ATR). VRC were ground and pressed into KBr dishes pellets before analysis from 400 to 4,000 cm^-1^.

### *In vitro* release

Nanofiber films containing 3 mg of VRC were immersed in 100 mL of 0.9% NaCl at 32°C with shaking at 50 rpm. The release medium was sampled (1 mL) for the drug assay and an equal amount of fresh medium was added at the times indicated. Before the HPLC assay, the samples were diluted with acetonitrile. A VRC solution was used as a control.

### Pharmacokinetics in rabbit tears

Eight rabbits were separated into a nanofiber-treated group and a VRC solution-treated group. VRC solution was prepared by dissolving VRC in a 0.9% (w/v) HPβCD solution to achieve a final 0.15% (w/v) drug concentration.

Rabbits were treated with a single administration of 40 μL of VRC solution or 1.5 mg of nanofibers, in which the drug dose was equivalent to that of the VRC solution. Tear fluid samples were collected at 0.25, 0.5, 0.75, 1.0, 2.0, 3.0, 4.0, 6.0, 8.0, 12.0, and 24.0 h after administration in the nanofiber group and at 0.25, 0.5, 0.75, 1.0, 2.0, 3.0, 4.0, 6.0, and 8.0 h after administration in the VRC solution group, using filter papers, which were weighed before use and gently inserted into the inferolateral cul-de-sac of the eye, close to the middle of the eye, for 30 seconds. After collection, papers were weighed again. Then, the papers were dried under stream of nitrogen gas and vortexed with 500 μL of methanol for 5 min. After centrifugation (12,000 rpm) for 10 min, the organic phase was transferred to another tube and evaporated to dryness. The residue was reconstituted with 50 μL methanol. The drug concentration in each sample was determined using HPLC.

### Ocular irritation test

An ocular irritation test (Draize eye test) was used to evaluate the ocular tolerance of the nanofibers.

Eight rabbits were randomly divided into two groups. One eye of the rabbit was treated with the VRC solution or VRC nanofibers, while the untreated eye functioned as a control. A successive administration once a day for 7 days was performed at the same dosages as those used in the pharmacokinetic study. The ocular irritation was observed at 1, 8 and 24 h after administration. The animals’ discomfort and symptoms in the conjunctiva, cornea, and lids were macroscopically examined using the Acute Eye Irritation/Corrosion scoring system established by the 2012 Organization for Economic Cooperation and Development for ocular irritation testing (Table 1) [26].

At the end of the test, the rabbits were euthanized by injection of a lethal dose of pentobarbital into the marginal ear vein. The cornea tissues were collected and fixed by immersion in buffered formalin (pH 7.4), dehydrated and embedded in paraffin. Sections were stained with hematoxylin and eosin (H&E) to evaluate potential injury to the cornea.

**Table 1:** Grading of ocular irritation test.

Grade	Cornea	Conjunctiva	Discharge	Lids
0	No alternations	No alternations	No discharge	No swelling
1	Mild opacity	Mild hyperemia; mild edema	Mild discharge without moistened hair	Mild swelling
2	Intense opacity	Intense hyperemia; Intense edema; Hemorrhage	Intense discharge with moistened hair	Obvious swelling

### HPLC analysis of VRC

The drug content in the VRC nanofibers was quantified by dissolving them in 30% (v/v) ethanol. The solutions were analyzed using HPLC. Drug loading rate (DL) was calculated:

$$DL(\%)=\frac{M_{VRC}}{M_{NF}}\times 100\%$$

where *M*_*VRC*_ is the actual VRC content in the weighed quantity of nanofibers, and *M*_*NF*_ is the weighed quantity of nanofibers.

The HPLC system used was of the Agilent 1260 series (Agilent, USA) and detection conditions were as follows: Agilent ZORBAX SB-C_18_ column(250 mm × 4.60 mm, 5 μm); flow rate: 1.0 mL/min; the UV detector wavelength: 256 nm. The mobile phase was acetonitrile: water (46:54).

### Statistical analysis

Kinetica™ V.4.4 with a non-compartment model was utilized to calculate the pharmacokinetics parameters in rabbit tears. Statistical data were analyzed using a Student’s t-test and a *P*-value of 0.05 was considered to be significant.

## Results and Discussion

### VRC dissolved in HPβCD solution

**Fig 1:**
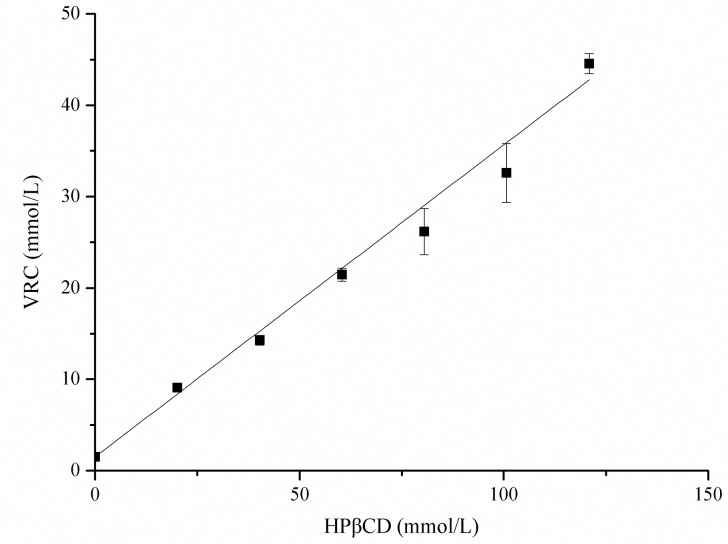
Solubility diagram for VRC in HPβCD at 37°C.

HPβCD, a cyclic oligosaccharide with an outer hydrophilic surface and a lipophilic cavity, is the most thoroughly studied cyclodextrin derivative. Numerous toxicological studies, pharmaceutical technological experiments and human clinical trials have been performed with HPβCD [27]. It is capable of forming inclusion complexes with VRC [28]. To increase the drug content in the nanofibers, we added HPβCD to the electrospinning solution. Fig 1 displays a linear increase in the solubility of VRC as a function of HPβCD concentration at 37°C, which displays a typical A_L_ type, according to Higuchi and Connors, indicating the formation of soluble complexes of a 1:1 stoichiometry [25]. The apparent stability constant (*Kc*) value was estimated to be 320 L/mol, according to the following equation [29]:

$$Kc=\frac{slop}{C_{0}\times (1-slop)}$$

where the slop was calculated by linear regression of the profile and C_0_ was the solubility of VRC in H_2_O. Compared with sulfobutylether-β-cyclodextrin (SBE-β-CD) used in VRC injections [30], HPβCD bears no ionizable groups, which results in a low conductivity, facilitating the preparation of nanofibers. Moreover, the solubilization capacity of HPβCD is comparable with that of SBE-β-CD.

### Fabrication of VRC-loaded nanofibers

**Fig 2:**
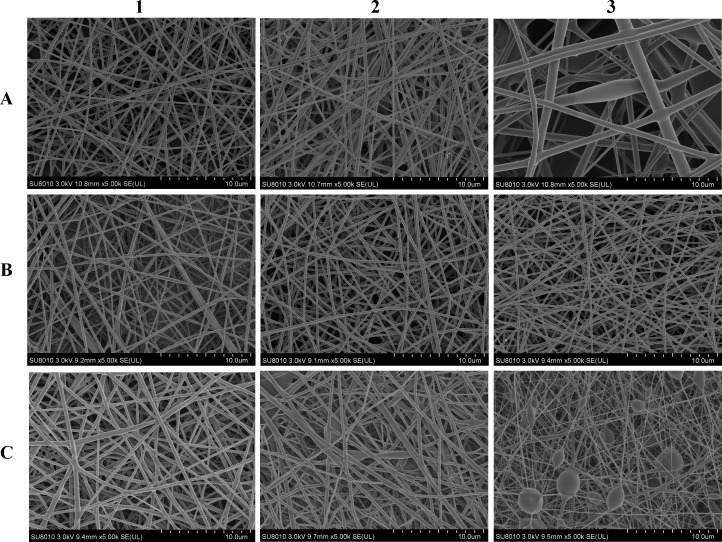
SEM images (×5000) of nanofibers fabricated by spinning solutions containing A1 6% PVA. A2 8% PVA. A3 10% PVA. B1 4% HPβCD, 8% PVA. B2 6% HPβCD, 8% PVA. B3 8% HPβCD, 8% PVA. C1 0.5% VRC, 4% HPβCD, 8% PVA (F1). C2 0.7% VRC, 6% HPβCD, 8% PVA (F2). C3 0.8% VRC, 8% HPβCD, 8% PVA (F3).

To prepare the nanofibers, the PVA concentration was first optimized. The blank nanofibers were fabricated using PVA with varied concentrations (Fig 2A1-A3). Bead-free and smooth fibers could be prepared using PVA solutions below 8% (w/v). The diameters were 204±23 nm, 210±31 nm, and 554±175 nm for fibers electrospun using 6%, 8% and 10% (w/v) PVA solutions, respectively. When the PVA concentration increased to 10%, fiber aggregations and fibers heterogeneity were observed and mean fiber diameters significantly increased. This was attributed to the increase in the viscosity of the spinning solution. The electrostatic field force was not enough to stretch the polymer to a uniform structure. Considering its higher spinning efficiency, we chose an 8% PVA solution for further investigation. Subsequently, varied amounts of HPβCD were added. Incorporation of HPβCD in the nanofibers did not alter the fiber morphology (Fig 2B1-B3). Inclusion of 4%, 6% and 8% (w/v) HPβCD in the spinning solution resulted in fiber diameters of 197±32nm, 212±32 nm, and 202±26 nm, respectively.

HPβCD could increase the solubility of VRC, and the enhancement was dependent on HPβCD concentration (Fig 1). To increase the drug content in the nanofibers, different amounts of HPβCD in conjunction with different levels of VRC were added to the spinning solution. Increasing the amounts of HPβCD and VRC resulted in an increase in the diameter of the drug loaded nanofibers. Similar results have also been reported for herbal oil loaded nanofibers [31]. The diameter of the nanofibers (F1) was 307±31 nm (Fig 2C1). When the HPβCD concentration was changed to 6%, the VRC content increased to 0.7% (F2), and the resultant nanofiber size was 315±123 nm (Fig 2C2). A higher concentration of VRC (0.8%) with 8% HPβCD (F3), resulted in the formation of beaded fibers (Fig 2C3). The actual drug loading rates of F1, F2 and F3 were 3.7±0.05%, 4.6±0.01% and 4.5±0.01%, respectively, which are much higher than those of nanofibers without HPβCD (below 1%), which were measured in a preliminary study. The addition of HPβCD rendered the nanofibers suitable for clinical application. The obtained nanofibers were used in an *in vitro* release study.

**Fig 3:**
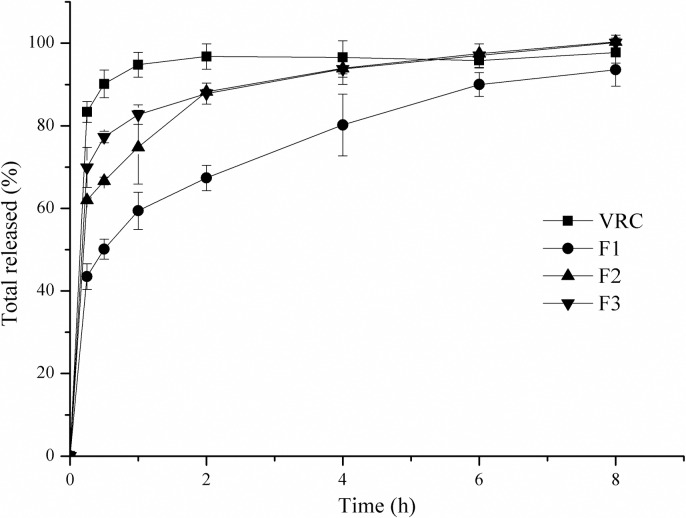
*In vitro* release of VRC electrospun nanofibers.

The dissolution profiles in Fig 3 show a significant delayed VRC release from the nanofibers compared with the VRC powder. More than 90% of the VRC powder was dissolved within 0.5 h. However, complete drug release was achieved after 2 h in the three nanofiber groups. Slower release from nanofibers with a higher PVA/HPβCD ratio was observed. The F1 fibers with a PVA/HPβCD ratio of 2:1 showed the slowest release among the nanofiber samples. VRC demonstrated sustained released from the nanofibers for 6 h. PVA is a linear, synthetic polymer traditionally used in pharmaceutical science because of its superior mechanical properties and high biocompatibility. This polymer can be included in tablets [32], microparticles [33], hydrogels [34], and patches [35] that swell and release entrapped drugs in a sustained way. The drug release rates are governed by the drug diffusion and polymer dissolution (surface erosion) [36]. Drug solubility, polymer molecular weight, polymer concentration and excipients are factors that affect the release profiles. In the present study, soluble HPβCD/VRC complex can readily diffuse from hydrogel matrix when the polymer concentration is relatively low. Inadequate PVA in F2 and F3 where the PVA/HPβCD mass ratios were 1.33:1 and 1:1 resulted in a faster release from the polymer matrix. All the fibers exhibited an initial burst release as a result of the immediate dissolution of the drug which became associated with the fiber surface during the electrospinning process [18]. F1 was chosen as the optimal formulation for *in vivo* evaluation, because of its defect free morphology, slow release, and relatively high drug loading.

### Characterization of the F1 VRC nanofibers

Cylindrically shaped continuous nanofibers without non-connected ‘tailed’ beads were formed (Fig 2C1). The average diameter of these fibers was approximately 300 nm. No drug crystals or other kinds of drug aggregates were observed on the surface of the fibers. It was presumed that the VRC was homogenously distributed in the spinning fibers owing to the increased solubility of the inclusion complex and interactions between PVA and VRC. The average thickness of the electrospun film was 235.6±11.2 μm. Taking into account the thickness of Ocusert^®^ (300 μm), few tolerance problems should be expected after application in the conjunctival sac [37].

**Fig 4:**
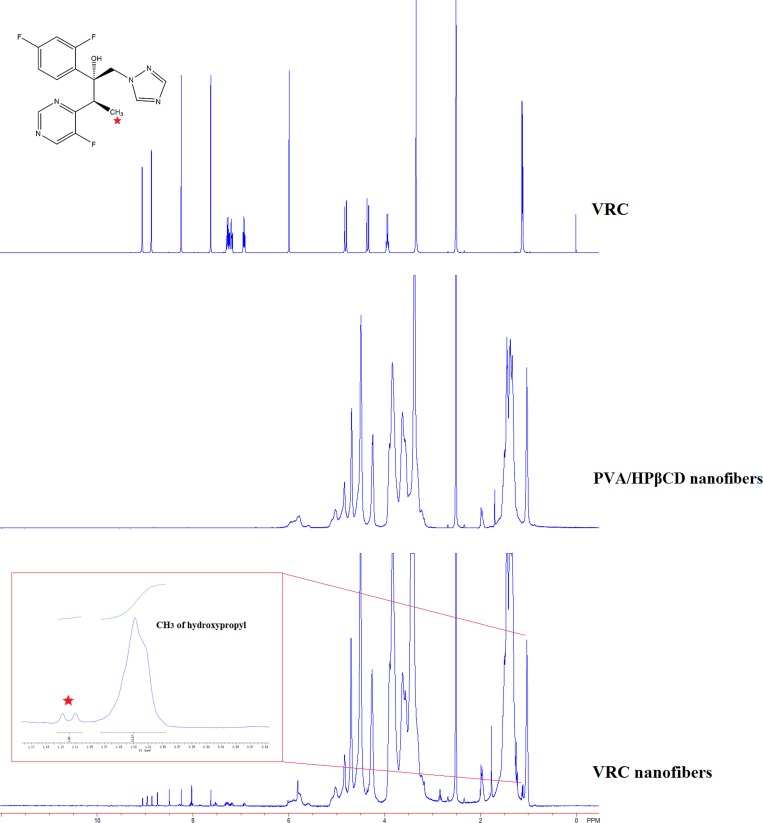
^1^H-NMR spectra of VRC, PVA/HPβCD nanofibers and VRC nanofibers. The insert was the integration of VRC peak (1.11ppm) and HPβCD peak (1.03 ppm).

Proton nuclear magnetic resonance (^1^H-NMR) was employed to further explore the molar ratio of HPβCD/VRC in nanofibers (Fig 4). The characteristic peaks of VRC ^1^H-NMR (d6-DMSO), δ (ppm): 9.05 (d, 1H), 8.24 (s, 1H), 8.08 (d, 1H), 7.63 (s, 1H), 6.95–6.91 (m, 3H), 2.51–2.5 (m, 2H), 1.11 (d, 3H) and HPβCD: 1.03 (s, 21H) were showed in the spectra of VRC nanofibers. The results indicated that VRC as well as HPβCD were electrospun in the nanofibers. In order to make the molar ratio calculations, the integration of HPβCD and VRC peaks were used. The molar ratio of HPβCD: VRC in nanofibers was calculated as 1:0.54 by taking the integration of the protons of HPβCD at 1.03 ppm and VRC at 1.11 ppm. According to the actual drug loading rate determined by HPLC, the molar ratio was calculated as 1:0.5. ^1^H-NMR results was in good consistence with HPLC results. Substantial amount of VRC was incorporated in nanofibers during the preparation and electrospinning.

**Fig 5:**
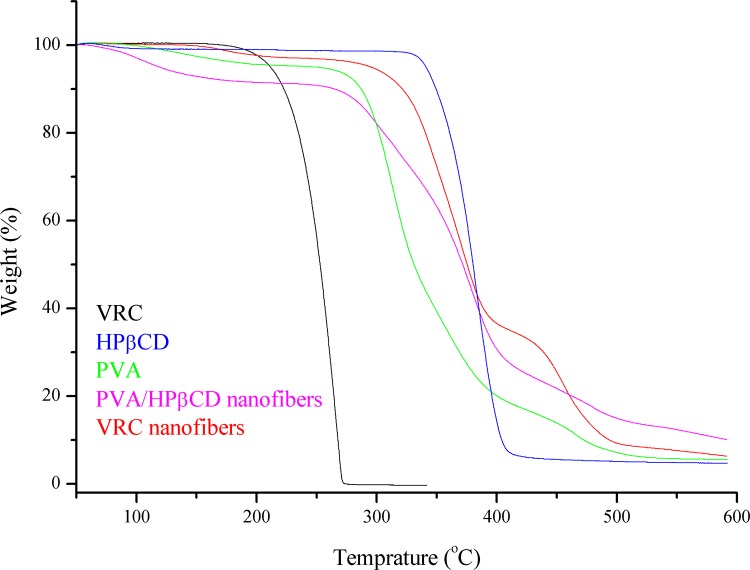
TGA thermograms of VRC, HPβCD, PVA, PVA/HPβCD nanofibers and VRC nanofibers.

TGA thermograms of VRC, HPβCD, PVA, PVA/HPβCD nanofibers and VRC nanofibers are given in Fig 5. The initial weight loss below 100°C was due to water loss. In the TGA curves of VRC and HPβCD, the major weight loss above 172.41°C and 320.69°C corresponded to the main thermal degradation of VRC and HPβCD. PVA powders exhibited three-step degradation: a small mass loss started below 185.17°C, a large loss took place in the temperature range of 258.28–408.96°C, and a medium mass loss took place in the range of 409–518.97°C. In the chase of PVA/HPβCD nanofibers, the three decomposition stages of PVA and thermal degradation behavior of HPβCD were observed. The second weight loss in VRC nanofibers were between 274.48 to 400.69°C. The shifting of thermal degradation onset of VRC from172.41°C to higher temperature suggested the existence of inclusion complex between HPβCD and VRC. Thermal stability of VRC was elevated in VRC nanofibers.

**Fig 6:**
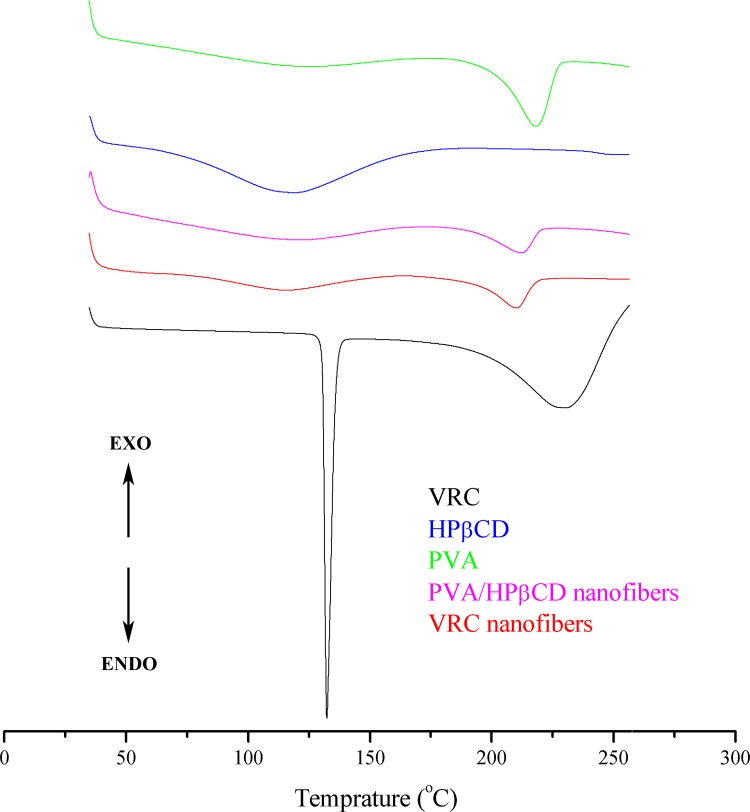
DSC thermograms of HPβCD, PVA, VRC, PVA/HPβCD nanofibers and VRC nanofibers.

DSC studies were undertaken to evaluate the physical state of VRC in the electrospun nanofibers and the thermograms are shown in Fig 6. Both HPβCD and PVA powders exhibited broad endothermic peaks between 60 and 160°C, and these peaks correspond to the dehydration of HPβCD and PVA. The melting temperatures (T_m_) for PVA was 218.42°C obtained from the DSC scan of PVA powders. Two endothermic peaks at around 120 and 215°C corresponding to the characteristic peaks of HPβCD and PVA were observed in PVA/HPβCD nanofibers. The thermogram of the VRC powder exhibited a melting point at 132.44°C whereas no melting point was observed for the VRC nanofibers, suggesting that VRC was incorporated into nanofibers in an amorphous state.

**Fig 7:**
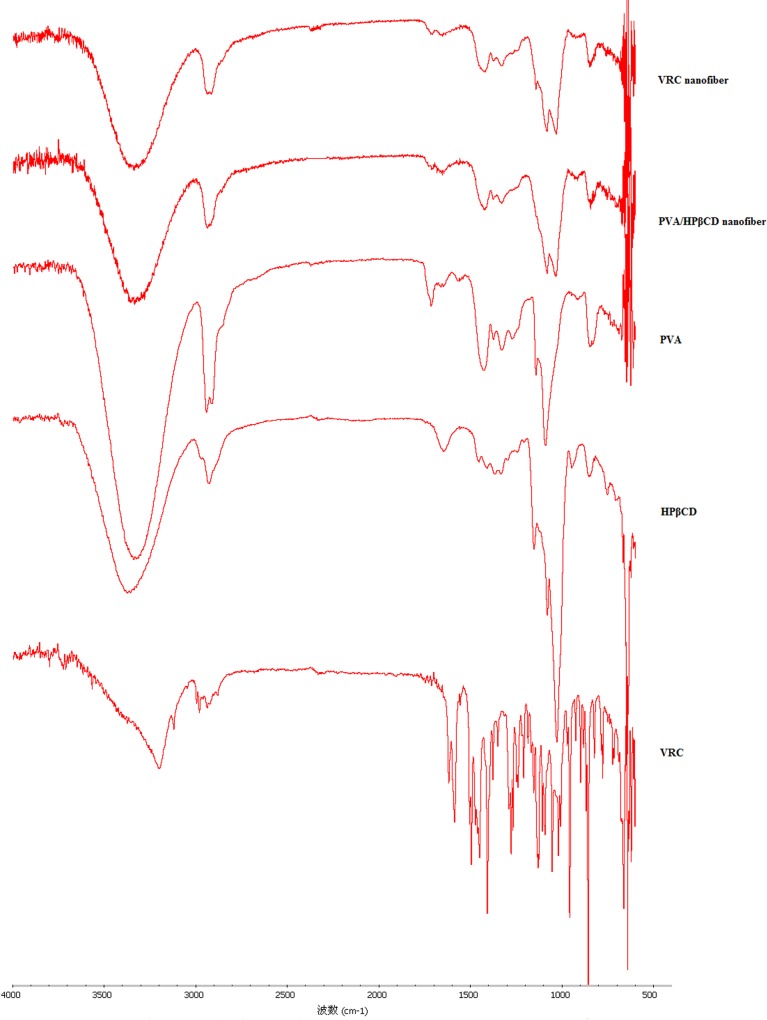
FT-IR spectra of VRC, PVA, HPβCD, PVA/HPβCD nanofibers and VRC electrospun nanofibers.

The FT-IR spectra of the VRC powder, PVA powder, HPβCD powder, PVA/HPβCD nanofiber and VRC nanofiber are displayed in Fig 7. The spectra of the PVA and HPβCD powders exhibited absorption peaks around 3350, 2930, and 1090 cm^-1^ that are attributed to the stretching vibrations of OH, CH and CO, respectively [38]. The VRC powder exhibited absorption peaks at 3200 cm^-1^ corresponding to the stretching vibrations of OH. The bands at 3000–2850, 1600–1400, and 1360–1250 cm^-1^ were assigned to the alkane CH, C = C aromatic and aryl C-N stretches [39]. The dominant absorption peaks observed in PVA and HPβCD were also observed in spectra of PVA/HPβCD nanofibers. VRC nanofibers displayed only the typical bands of PVA/HPβCD nanofibers, indicating that free VRC cannot be detected in the sample. This might actually indicate that VRC was completed included in the cyclodextrin cavity, as observed in VRC-cyclodextrin complexes [39]. There were interactions between VRC and HPβCD. The similar results were also obtained in itraconazole-cyclodextrin complex. The disappearance of FTIR bands was considered to be credible evidence for the presence of inclusion complexes [40, 41]. During our preparing of spinning solution, there was no purification process, and therefore there should be free VRC in the nanofibers [38]. However, VRC solubility in water is low. The insoluble VRC in the nanofibers is negligible. Therefore, the free VRC characteristic peak was not visible in the FI-IR spectra.

### Pharmacokinetics in rabbit tears

The majority of ophthalmic treatments are delivered as highly concentrated eye drops. They suffer from short precorneal residence time which is associated with low corneal drug absorption. Nasolachrymal drainage, lacrimal replacement and the relative impermeability of the corneal epithelial membrane lead to rapid and extensive loss of drugs from the precorneal area [42]. Therefore, in this study we developed nanofibers which formed three-dimensional networks with the ability to swell in aqueous solvents in a controlled manner [18], with the aim to achieve higher bioavailability and controlled ocular delivery. As an effective antifungal concentration [11, 12], we set the dose at 60 μg for the pharmacokinetics study. The established HPLC method for VRC determination met the *in vivo* analytical requirements. The low limit of quantification and low limit of detection of the method were 0.1 μg/mL and 0.03 μg/mL, respectively. The extraction recoveries at low, medium and high VRC levels were all greater than 90%. The standard curve for the drug assay in rabbit tears was Y = 418921X-21369 within 0.1–10 μg/mL VRC. The standard curve showed good linearity and the R^2^ was 0.9991.

**Fig 8:**
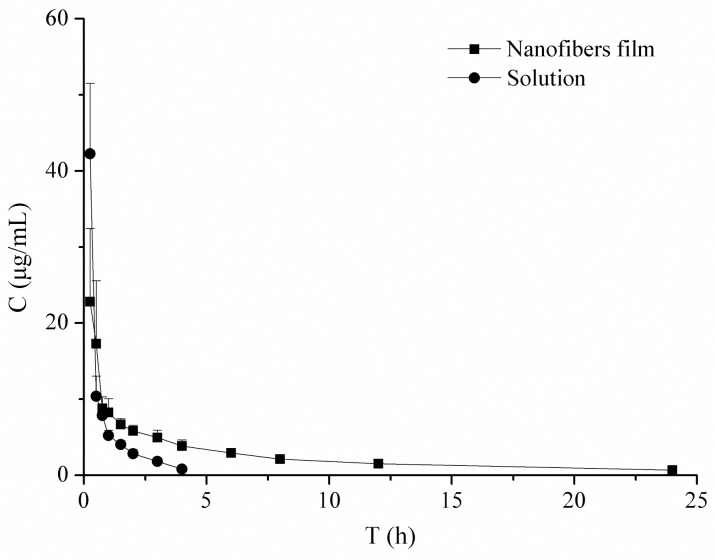
Concentration-time curves of VRC in tears after topical application of VRC solution or VRC-loaded nanofibers to the rabbit eye (n = 4, $\bar{x}$±*SD*).

The drug tear concentration-time profiles of the two formulations are shown in Fig 8. The VRC solution group achieved a higher drug concentration in the first 15 min, but it was eliminated rapidly. The drug was undetectable at 4 h. However, in the nanofiber group, the drug tear concentrations were all above 0.5 μg/mL until 24 h. The data were in accordance with the sustained drug release profiles of nanofibers *in vitro*. The calculated pharmacokinetic parameters are listed in Table 2, and the differences were mathematically evaluated by the calculation of *t*_*1/2*_ and *AUC*_*0-t*_. The nanofibers significantly prolonged the VRC half-life in tears, and increased the VRC bioavailability. Compared with the VRC solution, the relative bioavailability was 245%. The interaction of HPβCD with biological membranes has been reported to facilitate drug absorption in the tear film [43]. In addition to the prolonged residence time, a higher ocular drug bioavailability was achieved in the current study. Therefore, these nanofibers are expected to be effective in treating against fungal keratitis.

**Table 2:** Pharmacokinetic parameters of VRC in rabbit tears ($\bar{x}$±*SD*, n = 4).

	*AUC*_0→*t*_(*μg*⋅*h*⋅*mL*^−1^)	*t*_1/2_(*h*)
Solution	26.56±1.87	1.24±0.35
Nanofibers	64.69±10.51*	8.47±2.59*

* Compared with that of the solution group, *P*<0.05

### Ocular irritation test

PVA, which formed the nanofiber matrix, is a well-tolerated polymer type for ophthalmic drug delivery systems [24, 44]. HPβCD has also previously been shown to be safe in aqueous eye drop solutions, even at high concentrations [45]. In addition, the moderate thickness of the ocular nanofiber film means that it is unlikely to cause irritation.

**Fig 9:**
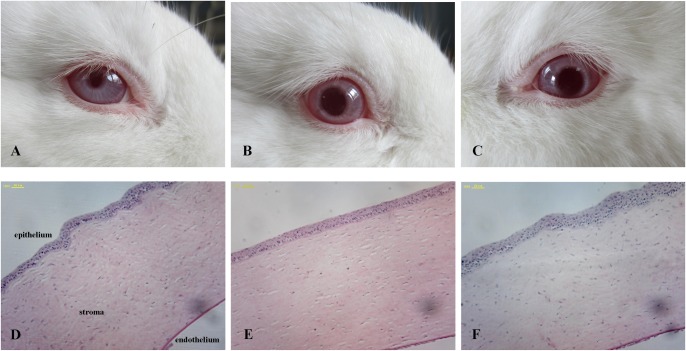
Results of irritation test at day 7 after the administration of VRC nanofibers or VRC solution. A-C Images of rabbit eyes. D-F Optical micrographs of corneal tissue (A, D Control. B, E VRC solution. C, F VRC nanofibers).

The Draize eye test results show that there was no ocular discomfort after the administration of the VRC nanofibers and the VRC solution (Fig 9A–9C). According to the Draize scoring standard, the eye irritation reaction scores were 0 for both groups. The H&E micrographs of the corneas (Fig 9D–9F) exhibit intact corneal structures and epithelia without histopathological abnormalities in the animals treated using the nanofibers. The results suggested that the VRC composited PVA/HPβCD nanofibers are a promising non-irritant system.

## Conclusion

In this research work, we successfully developed VRC incorporated PVA/HPβCD nanofibers for the treatment of fungal keratitis. The physicochemical characteristics, pharmacokinetics and ocular irritation of the optimized fibers were evaluated. SEM, TGA, DSC and FT-IR analyses suggest that VRC is present in an amorphous state in the nanofibers and there are interactions between VRC and HPβCD. Drug loading was increased because of the solubilization effect of HPβCD. A sustained release of VRC for 24 h was achieved. The half-life, *AUC*_0→*t*_, and bioavailability of VRC were all significantly increased in rabbit tears. Furthermore, few tolerance problems were observed after consecutive application for 7 days. It can be concluded that these nanofibers represent a promising delivery system to prolong the ocular delivery of VRC.
